# Long non-coding RNA TUG1/microRNA-187-3p/TESC axis modulates progression of pituitary adenoma via regulating the NF-κB signaling pathway

**DOI:** 10.1038/s41419-021-03812-7

**Published:** 2021-05-21

**Authors:** Rui Zhang, Fan Yang, Haitao Fan, Haocong Wang, Qinghao Wang, Jianxin Yang, Tao Song

**Affiliations:** 1 Department of Neurosurgery, Shandong Provincial Hospital Affiliated to Shandong First Medical University, 250021 Jinan, Shandong China; 2 Department of Neurosurgery, The People’s Hospital of Qingzhou, 262500 Qingzhou, Shandong China

**Keywords:** Cell biology, Diseases

## Abstract

The molecule mechanisms of long non-coding RNAs (lncRNAs) and microRNAs (miRNAs) in human diseases have been broadly studied recently, therefore, our research aimed to assess the effect of lncRNA taurine upregulated gene 1 (TUG1)/miR-187-3p/tescalcin (TESC) axis in pituitary adenoma (PA) by regulating the nuclear factor-kappa B (NF-κB) signaling pathway. We observed that TUG1 was upregulated in PA tissues and was associated with invasion, knosp grade and tumor size. TUG1 particularly bound to miR-187-3p. TUG1 knockdown inhibited cell proliferation, invasion, migration, and epithelial–mesenchymal transition, promoted apoptosis, and regulated the expression of NF-κB p65 and inhibitor of κB (IκB)-α in PA cells lines in vitro, and also inhibited tumor growth in vivo, and these effects were reversed by miR-187-3p reduction. Similarly, miR-187-3p elevation inhibited PA cell malignant behaviors and modulated the expression of NF-κB p65 and IκB-α in PA cells, and reduced in vivo tumor growth as well. TUG1 inhibition downregulated TESC, which was targeted by miR-187-3p. In conclusion, this study suggests that TUG1 sponges miR-187-3p to affect PA development by elevating TESC and regulating the NF-κB signaling pathway.

## Introduction

Arising from the adenohypophysial cells of the anterior pituitary^1^, pituitary adenoma (PA) constitutes at least 15% of intracranial neoplasms. The anterior pituitary is comprised of some hormone-producing cell types that can result in a rise to tumors, causing the heterogeneous group of neoplasms encompassed by the diagnosis of PA^2^. Microadenomas (diameter <10 mm) accounts for ~60% of PAs and macroadenomas (diameter >10 mm) accounts for ~40%, and these tumors can be ulteriorly grouped into functional (hormone secreting) and non-functional^3^. Although there is a high prevalence of PA in general people, these tumors are always benign and perform characteristics of differentiated pituitary cell function and premature proliferative arrest^4^. Previous studies have clarified that intrinsic and extrinsic factors can lead to the risks of PA^5–7^. Currently, treatments of PA include observation, medical therapy, surgery, and radiotherapy, which are depended on the nature and classification of the tumors^1^. However, these treatments are not available for many patients. Thus, the investigation to find a possible molecular target of PA might contribute to finding a novel treatment for this disease.

Long non-coding RNAs (lncRNAs) function as transcriptional activators and suppressors consistent with different chromatin modifiers^8^. The functions of many lncRNAs have been discussed in PA, such as lncRNA IFNG-AS1^9^ and lncRNA CCAT2^10^. LncRNA taurine upregulated gene 1 (TUG1), which is situated at chromosome 22q12, is initially verified as a part of photoreceptors and retinal development in murine retinal cells^11^. TUG1 has been revealed to regulate carcinogenesis in some malignancies, including malignant melanoma^12^ and glioma^13^. Although TUG1 exhibits differently high expression in tumors, its effect on PA remains largely unknown. Considering the oncogenic role of TUG1 in human brain tumors^14,15^, we speculated that it may participate in the progression of PA. LncRNAs are known to regulate the expression of microRNAs (miRNAs) through the decoy or sponge effect^16^. miRNAs are endogenous non-coding RNAs that play regulatory roles by complementary base pairing to 3′-untranslated region (3′-UTR) of protein-coding mRNA^17^. MiR-187-3p has been identified in human tumors, including hemangioma^18^ and hepatocellular carcinoma (HCC)^19^. Nevertheless, its role in PA remains to be further explored. Additionally, it has been reported that TUG1 sponges miR-29a in cholangiocarcinoma^20^ and sponges miR-498 in esophageal squamous cell carcinoma^21^. We found through bioinformatic prediction that there were binding sites between TUG1 and miR-187-3p, and this binding relationship has been scarcely investigated. Moreover, tescalcin (TESC) is a highly expressed gene in many cancer tissues and is thereby considered as an oncogene^22^. Although the impacts of TESC on malignancies such as renal cell carcinoma (RCC)^23^ and colorectal cancer (CRC)^24^ have been previously studied, its effect on PA as well as the target relation between TESC and miR-187-3p remains unexplored. Furthermore, nuclear factor-kappa B (NF-κB) is a nuclear transcription factor that is broadly expressed in the cytoplasm of higher eukaryotes^25^, and the NF-κB signaling pathway participates in human pituitary neoplasm^26^ and murine pituitary corticotroph tumor^27^. This research aimed to clarify the role of lncRNA TUG1/miR-187-3p/TESC axis in PA by regulating the NF-κB signaling pathway, and we hypothesized for the first time that TUG1 could competitively bind with miR-187-3p to regulate the development of PA by targeting TESC and modulating the NF-κB signaling pathway.

## Materials and methods

### Ethics statement

Written informed consents were acquired from all patients before this study. The protocol of this study was confirmed by the Ethic Committee of Shandong Provincial Hospital Affiliated to Shandong First Medical University (ethical number: 201851124). The protocol of animal experiments was approved by the Institutional Animal Care and Use Committee of Shandong Provincial Hospital Affiliated to Shandong First Medical University (ethical number: 201860913).

### Study subjects

Fifty-five PA specimens were harvested from PA patients (30 males and 25 females, aging 16–69 years, mean age of 51 years) that had accepted transsphenoidal surgery in Shandong Provincial Hospital Affiliated to Shandong First Medical University. According to the types of hypophyseal hormones, the cases were divided into 16 prolactin (PRL) tumors, 5 adrenocorticotrophic hormone (ACTH) tumors, 11 growth hormone (GH) tumors, and 23 non-functional tumors. Among the specimens, 10 cases <1 cm, 31 cases varied from 1 to 3 cm and 14 cases >3 cm in diameter. All the patients were diagnosed as PA by clinicopathological examination. Based on the standard of knosp classification^28^, the PA specimens were grouped in to I (10 cases), II (22 cases), III (17 cases), and IV (6 cases) stages. Specimens in III–VI stages were defined as the invasive group (24 cases) and those in 0–II stages were named as the non-invasive group (31 cases). The baseline data of these patients were recorded and patients with other malignancies or had accepted tumor therapies (drug therapy, radiotherapy, gamma knife, etc.) were excluded. In addition, 11 normal human anterior pituitary glands (the normal group) were collected from the donation process.

### Cell culture

PA cell lines (HP75 and GH3) were purchased from Beijing Zhongyuan Ltd. (Beijing, China). PA cell lines were cultured in media supplemented with 10% fetal bovine serum (FBS, Gibco, Thermo Fisher Scientific, Inc., CA, USA), streptomycin (100 μg/mL), and penicillin (100 units/mL). The above cells were cultured at 37 °C in a humidified atmosphere of 5% CO_2_.

### Cell grouping and transfection

The GH3 and HP75 cells were divided into seven groups: the Blank (no treatment), short hairpin RNA (sh)-negative control (NC) (transfection with NC of shRNA plasmid), sh-TUG1 (transfection with TUG1 shRNA plasmid), mimic NC (transfection with miR mimic NC), miR-187-3p mimic (transfection with miR-187-3p mimic), sh-TUG1 + inhibitor NC (transfection with TUG1 shRNA plasmid and miR inhibitor NC), and sh-TUG1 + miR-187-3p inhibitor (transfection with TUG1 shRNA plasmid and miR-187-3p inhibitor) groups. Oligonucleotides and plasmids used for transfection were obtained from GenePharma Co., Ltd. (Shanghai, China). All cell transfection was performed using Lipofectamine 3000 following the instructions of the manufacturer (Thermo Fisher Scientific).

### 3-(4,5-dimethyl-2-thiazolyl)-2,5-diphenyl-2-H-tetrazolium bromide (MTT) assay

Cells were seeded onto a 96-well plate at 1 × 10^4^ cells/mL. Each well was appended with 180 μL MTT solution when the cells were incubated for 24 h, 48 h, and 72 h. After incubated continuously for 2 h with the supernatant removed, each well was added with 150 μL dimethyl sulfoxide and shaken for 10 min. A microplate reader (Tecan M1000, Invitrogen) was used to measure the cell viability at 570 nm. The experiment was independently repeated for three times.

### Colony formation assay

Cells were trypsinized and made into cell suspension, which was seeded and incubated for 2–3 weeks. The incubation was stopped and the medium was discarded when the colonies could be observed by eyes. The cells were fixed, stained by Giemsa dye for 60 min and dried. The colonies were counted under a microscope. The experiment was independently repeated for three times.

### Flow cytometry

Cell cycle distribution and apoptosis were assessed as previously described^29^, and a flow cytometer (Biosciences, San Jose, CA) was used for the analysis. The experiment was independently repeated for three times.

### Transwell assay

Cells (3 × 10^4^) were seeded into an 8-μm pore membrane with or without Matrigel (1 : 8, Becton, Dickinson and Company, NJ, USA), and the basolateral chamber was set with medium with 10% FBS. Incubated for 24 h, cells on the apical layer were removed and those on the lower layer were fixed in Methanol for 20 min and stained with 0.1% crystal violet dye solution for 20 min. Five fields of view were randomly photographed and the transmembrane cells were counted. The experiment was independently repeated for three times.

### Reverse transcription quantitative polymerase chain reaction

Total RNA in tissues and cells was extracted by Trizol method (Invitrogen) and RNA was reversely transcribed into cDNA. The PCR was conducted by SYBR Green method. Glyceraldehyde phosphate dehydrogenase (GAPDH) was used as the loading control of TUG1 and TESC, and U6 was used as the loading control of miR-187-3p, Bcl-2, Bax, N-Cadherin, Vimentin, and E-Cadherin. The primers (Table 1) were synthetized by Genechem Co., Ltd. (Shanghai, China). The analysis was conducted by a PCR instrument (ABI 7500, ABI, CA, USA) and the data were analyzed by 2^−ΔΔCt^ method. The experiment was independently repeated for three times.

**Table 1:** Primer sequence.

Gene	Sequence
TUG1	F: 5′-TAGCAGTTCCCCAATCCTTG-3′
	R: 5′-CACAAATTCCCATCATTCCC-3′
MiR-187-3p	F: 5′-TGCAGGGTCCGAGGTATT-3′
	R: 5′-GCCGCTCGTGTCTTGTGTTGCAGC -3′
U6	F: 5′-CGCTTCGGCAGCACATATAC-3′
	R: 5′-TTCACGAATTTGCGTGTCAT-3′
Bcl-2	F: 5′-CTGTGCTGCTATCCTGC-3′
	R: 5′-TGCAGCCACAATACTGT-3′
Bax	F: 5′-CCCGAGAGGTCTTTTTCCGAG-3′
	R: 5′-CCAGCCCATGATGGTTCTGAT-3′
TESC	F: 5′-CCTACCATTCGCAAGGAGAA-3′
	R: 5′-TTCTCG ATGTGAGGGTTT CC-3′
N-Cadherin	F: 5′-AGGGGAGAGGTGCTCTACTG-3′
	R: 5′-GGGGTAATCCACACCACCTG-3′
Vimentin	F: 5′-TCCGCACATTCGAGCAAAGA-3′
	R: 5′-TGAGGGCTCCTAGCGGTTTA-3′
E-Cadherin	F: 5′-CGTCGAGCTCTTGACCGAAA-3′
	R: 5′-TCAAACACCTCCTGTCCTCT-3′
GAPDH	F: 5′-TGGGTGTGAACCATGAGAAG-3′
	R: 5′-GTGTCGCTGTTGAAGTCAGA-3′

*F* forward, *R* reverse, *TUG1* taurine upregulated gene 1, *miR-187-3p* microRNA-187-3p, *TESC* tescalcin, *GAPDH* glyceraldehyde phosphate dehydrogenase.

### Western blot analysis

Cells and tissues were lysed with 1 mL lysate plus 10 μL of PMSF (100 mM) for 30 min, and the lysate was centrifuged at 12,000 rpm for 5 min at 4 °C. The supernatant was placed in a 0.5-mL centrifuge tube and placed at −20 °C. Protein concentration was determined using the bicinchoninic acid method (Solarbio Science & Technology Co., Ltd., Beijing, China). The extracted proteins were conducted with 10% sodium dodecyl sulfate-polyacrylamide gel electrophoresis (Boster) and transferred onto membranes, which were blocked by 5% bovine serum albumin for 1 h and appended with primary antibodies TESC (1 : 1000, Santa Cruz Biotechnology Inc., CA, USA), NF-κB p65 (ab32536, 1: 2000), IκB-α (ab32518, 1 : 1000), and β-actin (ab8226, 1 : 5000, all from Abcam Inc., MA, USA), then incubated at 4 °C overnight. Afterwards, the membranes were supplemented with corresponding secondary antibody (Miaotong Biotechnology Co., Ltd., Shanghai, China) for 1-h incubation. The membranes were developed by enhanced chemiluminescent reagent and Bio-rad Gel Doc EZ imager (Bio-rad Laboratories, CA, USA), and the gray values of the protein bands were analyzed by Image J software (National Institutes of Health, MD, USA). The experiment was independently repeated for three times.

### Dual luciferase reporter gene assay

The bioinformatic website https://cm.jefferson.edu/rna22/Precomputed/ (or http://www.targetscan.org/vert_72/) was used to measure the binding sites of TUG1 and miR-187-3p (or binding sites of miR-187-3p and TESC). Luciferase reporter gene vectors (pGL3-Control vector, Promega, WI, USA) containing wild-type (Wt) or mutant (Mut) TUG1 and the 3′-UTR of Wt or Mut TESC were co-transfected with miR-187-3p mimic and mimic NC. After 48 h, dual luciferase reporter assay was performed based on the manufacturer’s instructions (Promega). The Renilla luciferase signal was normalized to the firefly luciferase signal for each individual analysis. The experiment was independently repeated for three times.

### RNA pull-down assay

The miR-187-3p, miR-187-3p-Mut, and NC probes were synthesized and biotinylated by GenePharma. The RNA pull-down assay was conducted using the Magnetic RNA-Protein Pull-Down Kit (Thermo Fisher) according to the manufacturer’s protocol. Cells were transfected with biotinylated miRNA and the M-280 streptavidin magnetic beads (Invitrogen) were used to incubate with cell lysates. Then, TUG1 expression was assessed using reverse transcription quantitative polymerase chain reaction (RT-qPCR). The experiment was independently repeated for three times.

### Subcutaneous tumorigenesis in nude mice

BALB/c nude mice (aging 6 weeks, weighing 18–20 g) were gained from the Laboratory Animal Center of Shandong University (Shandong, China). The mice were classified into 2 large groups and 14 small groups: the blank (injection of GH3 and HP75 cells without any treatment), sh-NC (injection of GH3 and HP75 cells stably transfected with NC of TUG1 shRNA plasmid), sh-TUG1 (injection of GH3 and HP75 cells stably transfected with TUG1 shRNA plasmid), mimic NC (injection of GH3 and HP75 cells stably transfected with miR mimic NC), miR-187-3p mimic (injection of GH3 and HP75 cells stably transfected with miR-187-3p mimic), sh-TUG1 + inhibitor NC (simultaneous injection of GH3 and HP75 cells stably transfected with TUG1 shRNA plasmid and miR inhibitor NC) and sh-TUG1 + miR-187-3p inhibitor (simultaneous injection of GH3 and HP75 cells stably transfected with TUG1 shRNA plasmid and miR-187-3p inhibitor) groups. Cell suspension (100 μL) was injected into the nude mice at the armpit of right forelimb of nude mice 0.3–0.5 cm away from the back. After 24-day incubation, the mice were euthanized by CO_2_ with the tumor tissues collected for subsequent experiment.

### Hematoxylin–eosin staining

The xenografts were washed with normal saline and fixed in 4% paraformaldehyde for 30–50 min, and then were washed, dehydrated, permeabilized, waxed, embedded, and sliced. After that, the tissue sections were put on the slide, dried in a 45 °C incubator, dewaxed, soaked in gradient ethanol, and washed with distilled water for 5 min. Stained with hematoxylin for 5 min, the sections were washed with running water for 3 s, differentiated with 1% hydrochloric acid ethanol for 3 s, and stained with 5% eosin solution for 3 min. Subsequently, the sections were dehydrated, permeabilized, sealed, and observed under a microscope.

### Statistical analysis

The experimental data and image preprocessing were analyzed by SPSS 20 statistical software (IBM, USA) and GraphPad Prism7.0 software (La Jolla, USA). All data were shown as mean ± standard deviation (SD). Student’s *t* test was used to analyze differences between the two groups, and the differences between multiple groups were compared by one-way ANOVA with a post hoc Tukey test. *P* value < 0.05 was indicative of statistically significant difference.

## Results

### Expression of TUG1 in PA and its correlation with tumorigenesis

**Fig. 1:**
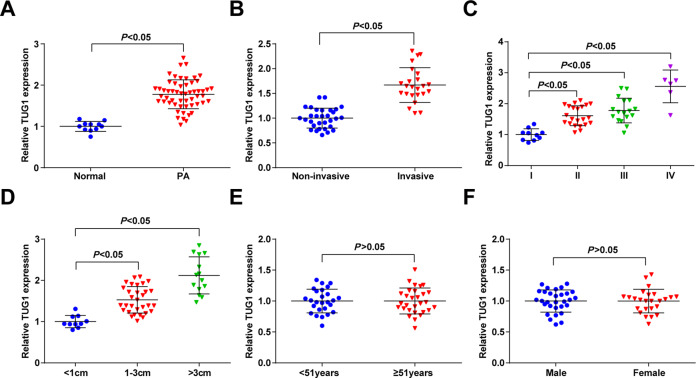
Expression of TUG1 in PA and its correlation with tumorigenesis. **A** TUG1 expression in PA tissues was detected using RT-qPCR; **B** TUG1 expression in invasive and non-invasive PA tissues was detected using RT-qPCR; **C** TUG1 expression in PA tissues with different knosp grades was detected using RT-qPCR; D, TUG1 expression in PA tissues with different tumor sizes was detected using RT-qPCR E, TUG1 expression in PA tissues from PA patients with different ages was detected using RT-qPCR; F, TUG1 expression in PA tissues from PA patients with different genders was detected using RT-qPCR; *n* = 11 in the normal group and *n* = 55 in the PA group. Data are presented as mean ± SD.

TUG1 has been found to be upregulated in multiple cancers and participate in the proliferation, migration, invasion, and drug-resistance of cancer cells^30–32^. Moreover, it has been reported that TUG1 was up-expressed in glioblastoma^15^. We detected the expression of TUG1 in PA tissues and normal pituitary tissues using RT-qPCR to verify its abnormal expression in PA, and the results suggested that versus the normal pituitary tissues, the expression of TUG1 was heightened in PA tissues (Fig. 1A). Based on this finding, we further analyzed the association between TUG1 and PA tumorigenesis. The PA tissues were classified into the invasive group and the non-invasive group. It was found that TUG1 expression in the invasive group was higher than the non-invasive group (Fig. 1B). We further determined the relations of TUG1 expression with knosp grade and tumor size, and the outcomes indicated that the higher knosp grade suggested a higher expression of TUG1 in PA tissue (Fig. 1C) and the larger tumors had a higher expression of TUG1 (Fig. 1D), while TUG1 expression was not changed with age and gender (Fig. 1E, F).

### TUG1 binds to miR-187-3p, and TESC is targeted by miR-187-3p

**Fig. 2:**
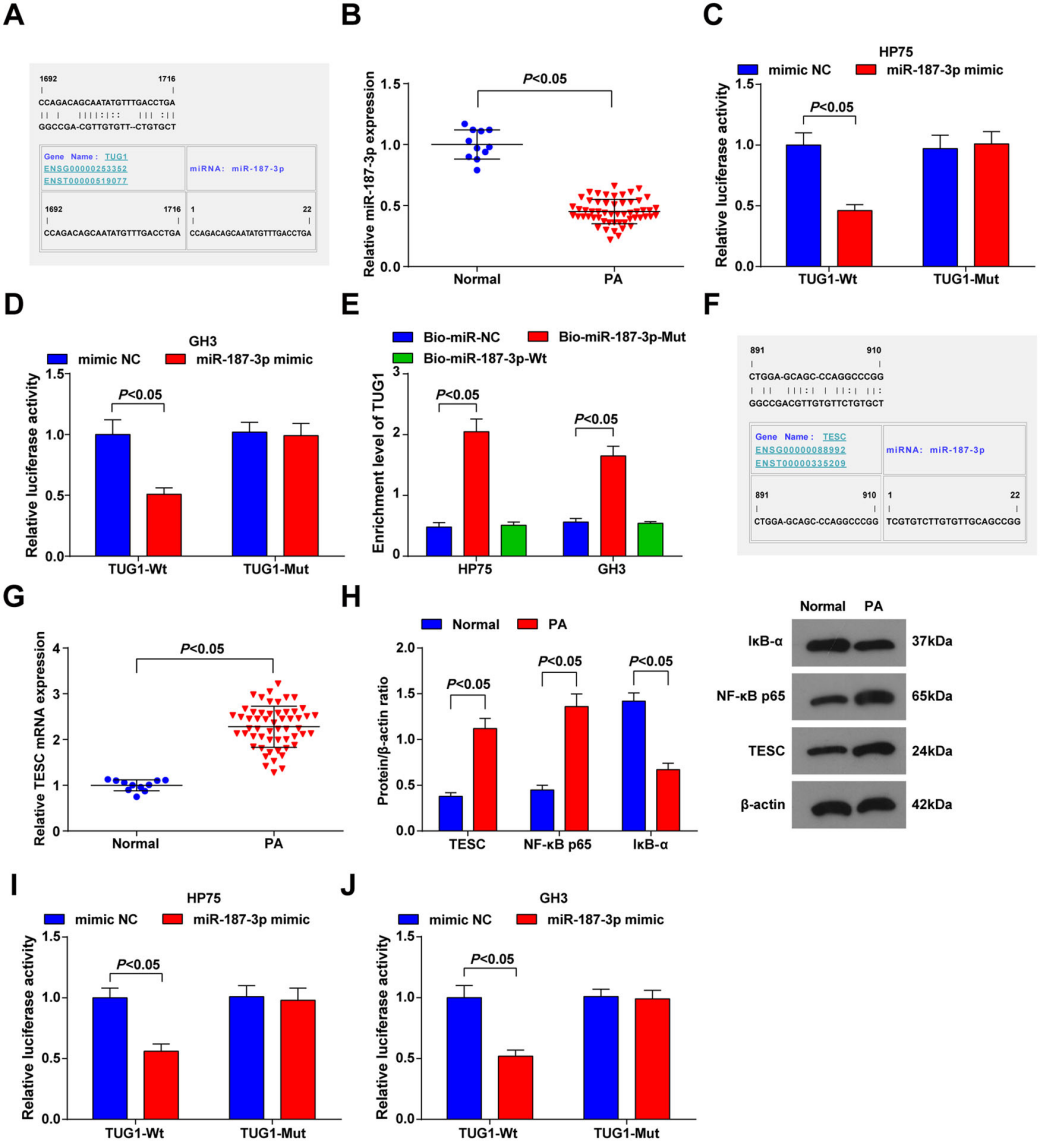
TUG1 binds to miR-187-3p, and TESC is targeted by miR-187-3p. **A** binding sites of TUG1 and miR-187-3p were predicted by a bioinformatic website; **B** miR-187-3p expression in PA tissues was detected using RT-qPCR; **C** regulatory relation between TUG1 and miR-187-3p in HP75 cells was confirmed by dual luciferase reporter gene assay; **D** regulatory relation between TUG1 and miR-187-3p in GH3 cells was confirmed by dual luciferase reporter gene assay; **E** regulatory relation between TUG1 and miR-187-3p in GH3 and HP75 cells was confirmed by RNA pull-down assay; **F** binding sites of miR-187-3p and TESC were predicted by a bioinformatic website; **G** TESC mRNA expression in PA tissues was detected using RT-qPCR; **H** expression levels of TESC, NF-κB p65 and IκB-α in PA tissues were detected using western blot analysis; **I** target relation between miR-187-3p and TESC in HP75 cells was confirmed by dual luciferase reporter gene assay; **J** target relation between miR-187-3p and TESC in GH3 cells was confirmed by dual luciferase reporter gene assay. *n* = 11 in the normal group and *n* = 55 in the PA group. Repetitions = 3, Data are presented as mean ± SD.

LncRNAs are known to function as competing endogenous RNA (ceRNAs) during tumourigenesis. The ceRNAs can interact with functional miRNAs to regulate gene expression. It was predicted at the online analysis website RNA22 (https://cm.jefferson.edu/rna22/Precomputed/) that there existed particular binding region between the sequences of TUG1 and miR-187-3p (Fig. 2A). Then, we detected the expression of miR-187-3p in PA tissues. The results showed that miR-187-3p was downregulated in PA tissues versus normal pituitary tissues (Fig. 2B). Subsequently, TUG1 luciferase reporter plasmids with Wt and predicted mutant sites for miR-187-3p were constructed. We found that miR-187-3p mimic repressed the luciferase activity of the Wt plasmid whereas did not affect the luciferase activity of the mutant plasmid (Fig. 2C). Moreover, the results of RNA pull-down assay illustrated that TUG1 was pulled down by the target oligos (Fig. 2D). These data implied that TUG1 is a sponge for miR-187-3p.

RNA22 (https://cm.jefferson.edu/rna22/Precomputed/0) was also used to identify the potential targets of miR-187-3p. It was found that TESC may be a target of miR-187-3p (Fig. 2E). The expression of TESC in tissues was determined and we found that TESC was upregulated in PA tissue versus normal pituitary tissues (Fig. 2F). The results of dual luciferase reporter gene assay suggested that (Fig. 2G) the co-transfection of TESC-Wt and miR-187-3p mimic decreased the luciferase activity of the cells, while the luciferase activity was not broadly changed after the PA cells co-transfected with TESC-Mut and miR-187-3p mimic, showing a targeting relationship between miR-187-3p and TESC.

A study has revealed that TESC promoted NF-κB signaling pathway, thus facilitating tumorigenesis^33^. We detected the expression NF-κB p65 and IκB-α in PA tissues, and it came out that the expression levels of NF-κB p65 were increased, while that of IκB-α were suppressed in PA tissues versus normal pituitary tissues (Fig. 2F).

### Inhibited TUG1 or elevated miR-187-3p restrains proliferation and promotes cell cycle progression of PA cells

The viability, colony formation ability, and cell cycle distribution of PA cells were determined respectively using MTT assay, colony formation assay, and flow cytometry to explore the roles of TUG1 or miR-187-3p on PA cells.

**Fig. 3:**
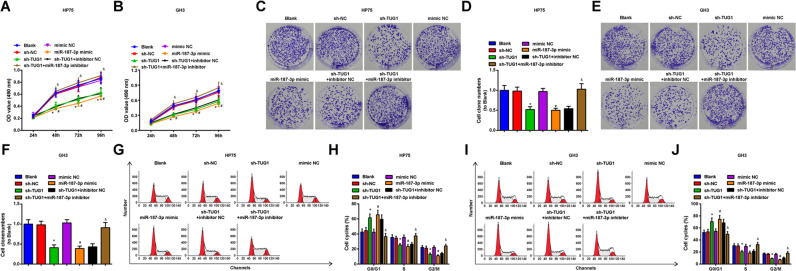
Inhibited TUG1 or elevated miR-187-3p restrains proliferation and promotes cell cycle progression of PA cells. **A** viability of HP75 cells was assessed by MTT assay; **B** viability of GH3 cells was assessed by MTT assay; **C** colony formation ability of HP75 cells was measured by colony formation assay; **D** statistical results of colony formation ability of HP75 cells; **E** colony formation ability of GH3 cells was measured by colony formation assay; **F** statistical results of colony formation ability of GH3 cells; **G** cell cycle distribution of HP75 cells was determined by flow cytometry; **H** statistical results of cell cycle distribution of HP75 cells; **I** cell cycle distribution of GH3 cells was determined by flow cytometry; **J** statistical results of cell cycle distribution of GH3 cells; repetitions = 3. Data are presented as mean ± SD. *P < 0.05 vs the sh-NC group, #*P* < 0.05 vs the mimic NC group, & *P* < 0.05 vs the sh-TUG1 + inhibitor NC group.

MTT assay was used to evaluate the viability of GH3 and HP75 cells, and the results (Fig. 3A, B) revealed that silenced TUG1 or elevated miR-187-3p repressed the cell viability, while the effect of silenced TUG1 on cell viability was reversed by miR-187-3p inhibition.

The colony formation ability of GH3 and HP75 cells was determined using colony formation assay and we found that (Fig. 3C–F) TUG1 downregulation or miR-187-3p upregulation restrained the colony formation ability of cells, and the impact of TUG1 downregulation was abolished by miR-187-3p reduction.

Flow cytometry was employed to assess the cell cycle distribution of GH3 and HP75 cells, and the outcomes (Fig. 3G–J) reflected that TUG1 reduction or miR-187-3p elevation arrested cells at G0/G1 phase, while the effect of TUG1 reduction on cell cycle distribution was abrogated by miR-187-3p suppression.

The above findings suggest that TUG1 promotes proliferation in PA cell lines by sponging miR-187-3p.

### Inhibited TUG1 or elevated miR-187-3p accelerates apoptosis of PA cells

To investigate the effect of TUG1 or miR-187-3p on PA cells, we detected the apoptosis of PA cells using flow cytometry and assessed expression of apoptotic factors in PA cells using RT-qPCR.

**Fig. 4:**
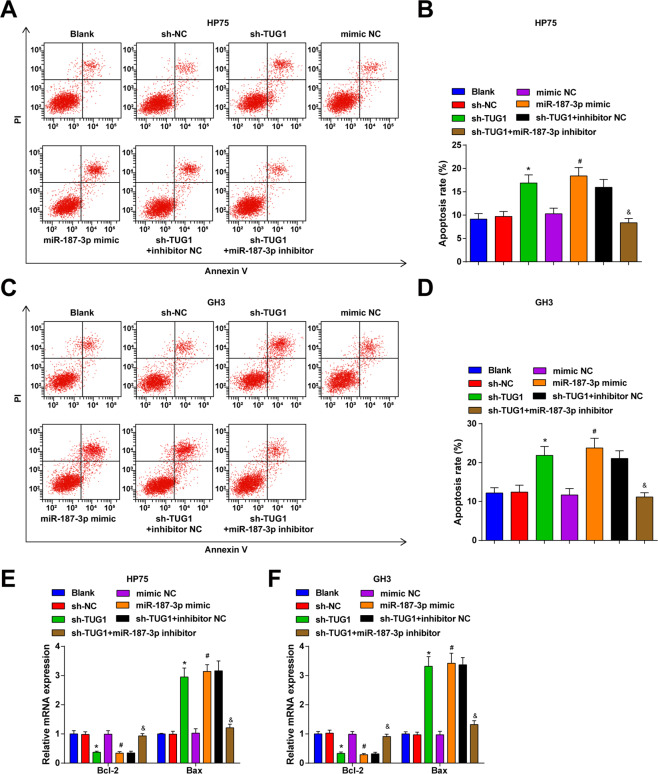
Inhibited TUG1 or elevated miR-187-3p accelerates apoptosis of PA cells. **A** apoptosis of HP75 cells was evaluated by flow cytometry; **B** statistical results of apoptosis of HP75 cells; **C** apoptosis of GH3 cells was evaluated by flow cytometry; **D** statistical results of apoptosis of GH3 cells; **E** mRNA expression of apoptotic proteins Bcl-2 and Bax in HP75 cells was detected using RT-qPCR; **F** mRNA expression of apoptotic proteins Bcl-2 and Bax in GH3 cells was detected using RT-qPCR; repetitions = 3. Data are presented as mean ± SD. *P < 0.05 vs the sh-NC group, #*P* < 0.05 vs the mimic NC group, &*P* < 0.05 vs the sh-TUG1 + inhibitor NC group.

The apoptosis rate of GH3 and HP75 cells was obtained from flow cytometry (Fig. 4A–D) and it came out that inhibited TUG1 or upregulated miR-187-3p promoted the apoptosis of GH3 and HP75 cells, and reduced miR-187-3p reversed the promotive role of inhibited TUG1 in cell apoptosis.

The mRNA expression of apoptotic proteins (Bax and Bcl-2) in GH3 and HP75 cells was detected by RT-qPCR. It was found that TUG1 silencing or miR-187-3p elevation increased Bax mRNA expression while decreased Bcl-2 mRNA expression in GH3 and HP75 cells; the alterations on Bax and Bcl-2 expression induced by TUG1 silencing could be reversed by miR-187-3p inhibition (Fig. 4E, F). These data indicated that TUG1 inhibited apoptosis in PA cell lines by sponging miR-187-3p.

### Inhibited TUG1 or elevated miR-187-3p decelerates migration and invasion of PA cells

We detected the effect of TUG1 or miR-187-3p on the migration and invasion abilities of GH3 and HP75 cells to evaluate whether TUG1 or miR-187-3p contributes to the metastasis of PA.

**Fig. 5:**
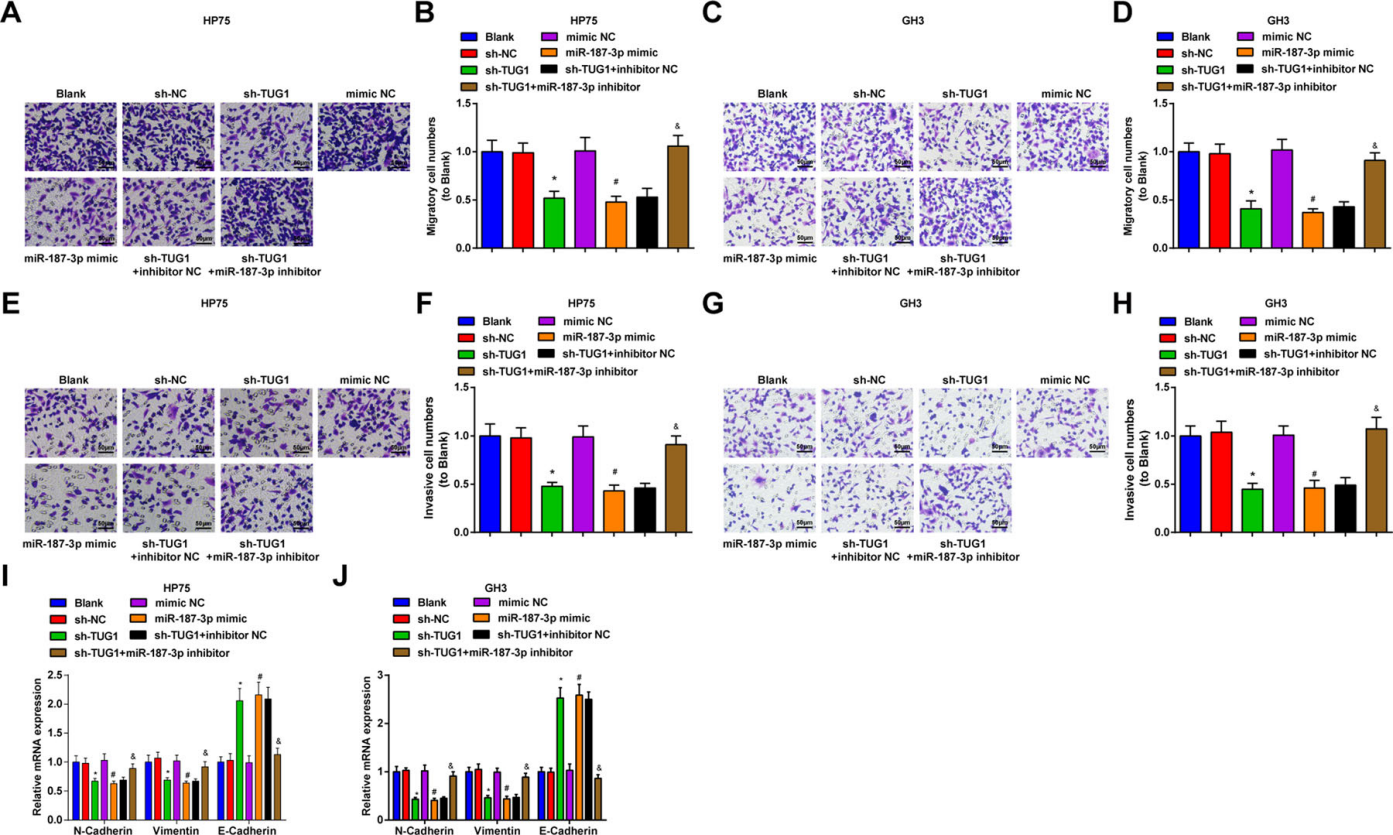
Inhibited TUG1 or elevated miR-187-3p decelerates migration and invasion of PA cells. **A** migration ability of HP75 cells was assessed by Transwell assay; **B** statistical results of migration ability of HP75 cells; **C** migration ability of GH3 cells was assessed by Transwell assay; **D** statistical results of migration ability of GH3 cells; **E** invasion ability of HP75 cells was assessed by Transwell assay; **F** statistical results of invasion ability of HP75 cells; **G** invasion ability of GH3 cells was assessed by Transwell assay; **H** statistical results of invasion ability of GH3 cells; **I** mRNA expression of EMT-related factors N-Cadherin, E-Cadherin, and Vimentin in HP75 cells was detected using RT-qPCR; J, mRNA expression of EMT-related factors N-Cadherin, E-Cadherin, and Vimentin in GH3 cells was detected using RT-qPCR; repetitions = 3. Data are presented as mean ± SD. *P < 0.05 vs the sh-NC group, #*P* < 0.05 vs the mimic NC group, &*P* < 0.05 vs the sh-TUG1 + inhibitor NC group.

Transwell assay was used to determine the migration and invasion abilities of GH3 and HP75 cells (Fig. 5A–H), and we observed that the migration and invasion abilities of cells were restricted by TUG1 silencing or miR-187-3p amplification, while the role of TUG1 silencing was reversed by miR-187-3p downregulation. The expression of EMT-related proteins (N-Cadherin, E-Cadherin, and Vimentin) was determined using RT-qPCR and the results showed that reduced TUG1 or elevated miR-187-3p promoted the mRNA expression of E-Cadherin and suppressed the mRNA expression of N-Cadherin and Vimentin in GH3 and HP75 cells, while these effects of reduced TUG1 were abolished by downregulated miR-187-3p (Fig. 5I, J). It could be concluded that TUG1 promotes invasion and migration in PA cell lines by sponging miR-187-3p.

### TUG1 or miR-187-3p affects the NF-κB signaling pathway

**Fig. 6:**
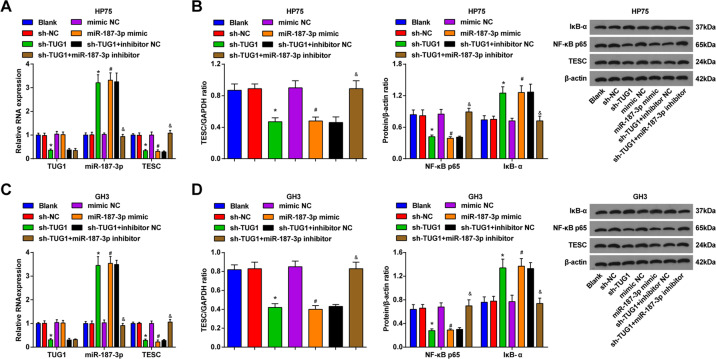
TUG1 or miR-187-3p affects the NF-κB signaling pathway. **A** expression levels of TUG1, miR-187-3p, and TESC in HP75 cells were assessed using RT-qPCR; **B** protein expression of TESC, NF-κB p65, and IκB-α in HP75 cells was assessed using western blot analysis; **C** expression levels of TUG1, miR-187-3p, and TESC in GH3 cells were determined using RT-qPCR; **D** protein expression of TESC, NF-κB p65, and IκB-α in GH3 cells was evaluated using western blot analysis; repetitions = 3. Data are presented as mean ± SD. *P < 0.05 vs the sh-NC group, #*P* < 0.05 vs the mimic NC group, &*P* < 0.05 vs the sh-TUG1 + inhibitor NC group.

Expression levels of TUG1, miR-187-3p, TESC, NF-κB/p65, and IκB-α in PA cells were assessed and the results (Fig. 6A–D) suggested that TUG1 knockdown downregulated TUG1, TESC, and NF-κBp65, while upregulated miR-187-3p and IκB-α; miR-187-3p elevation inhibited expression of TESC and NF-κBp65 and promoted expression of miR-187-3p and IκB-α; the alterations on expression levels of miR-187-3p, TESC, NF-κB/p65, and IκB-α that induced by TUG1 knockdown were reversed by inhibited miR-187-3p.

### Inhibited TUG1 or elevated miR-187-3p restrains tumor growth of PA in vivo

**Fig. 7:**
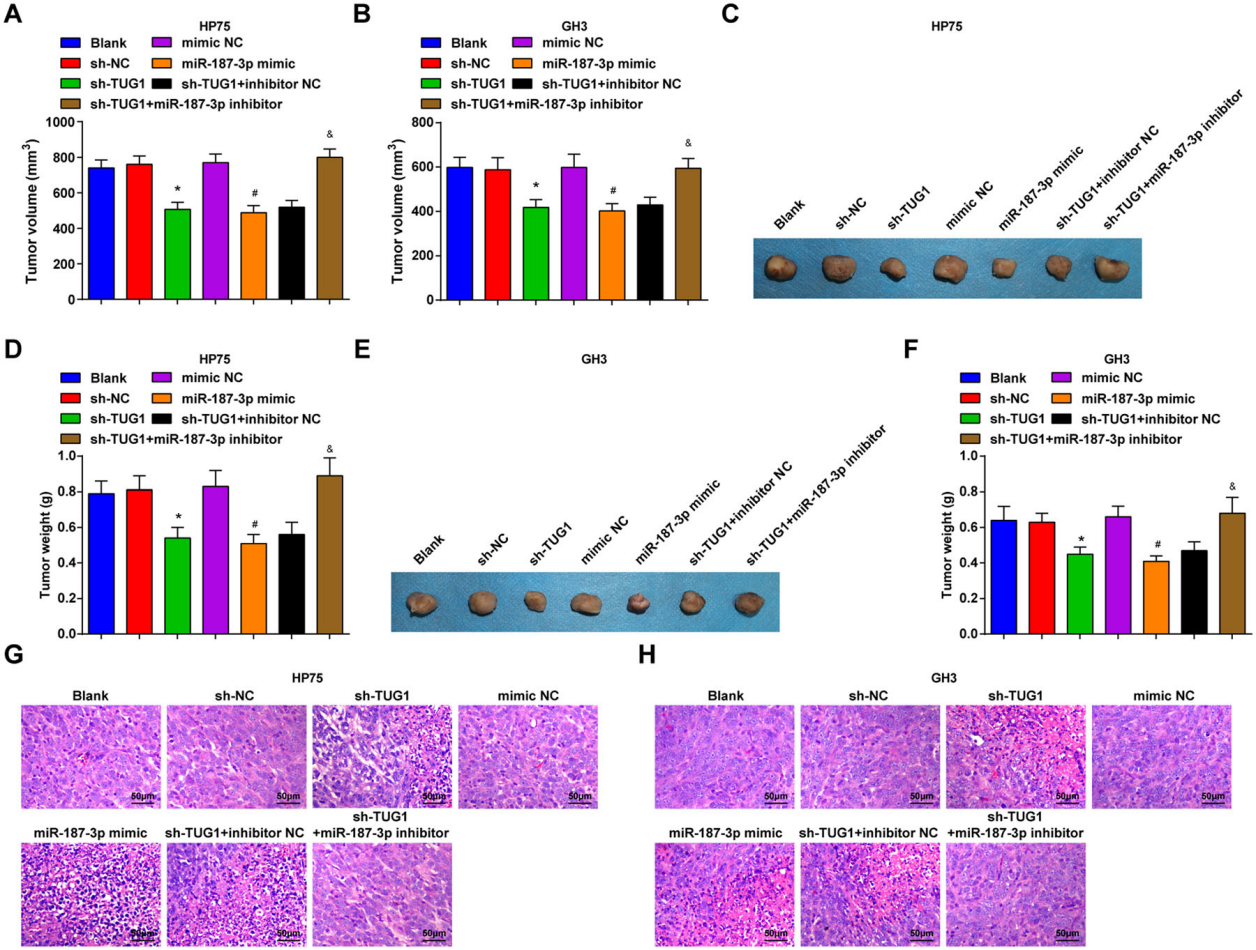
Inhibited TUG1 or elevated miR-187-3p restrains tumor growth of PA in vivo. **A** Tumor volume of nude mice that have been injected with HP75 cells on the 24th day; **B** Tumor volume of nude mice that have been injected with GH3 cells on the 24th day; **C** representative tumors figures of nude mice that have been injected with HP75 cells on the 24th day; **D** tumor weight of nude mice that have been injected with HP75 cells; **E** representative tumors figures of nude mice that have been injected with GH3 cells on the 24th day; **F** tumor weight of nude mice that have been injected with GH3 cells; **G** pathological changes in xenografts from nude mice after injection of HP75 cells observed using HE staining; **H** pathological changes in xenografts from nude mice after injection of GH3 cells observed using HE staining; *n* = 5 mice. Data are presented as mean ± SD. *P < 0.05 vs the sh-NC group, #*P* < 0.05 vs the mimic NC group, &*P* < 0.05 vs the sh-TUG1 + inhibitor NC group.

We established a PA xenograft model to measure the role of TUG1 or miR-187-3p in vivo. The xenografts in nude mice were shown in Fig. 7A–F. It was observed that mice accepted the injection of cell suspension treated with TUG1 silencing or miR-187-3p elevation had repressed tumor weight and volume, and miR-187-3p inhibition abrogated the impact of TUG1 repression.

Hematoxylin–eosin (HE) staining was employed to observe the pathological changes in the xenografts from nude mice, and the results implied that a loose arrangement and a large necrotic region existed in xenografts from nude mice that accepted the injection of cell suspension treated with TUG1 silencing or miR-187-3p elevation, while the effect of TUG1 knockdown was abolished by miR-187-3p reduction (Fig. 7G, H).

## Discussion

**Fig. 8:**
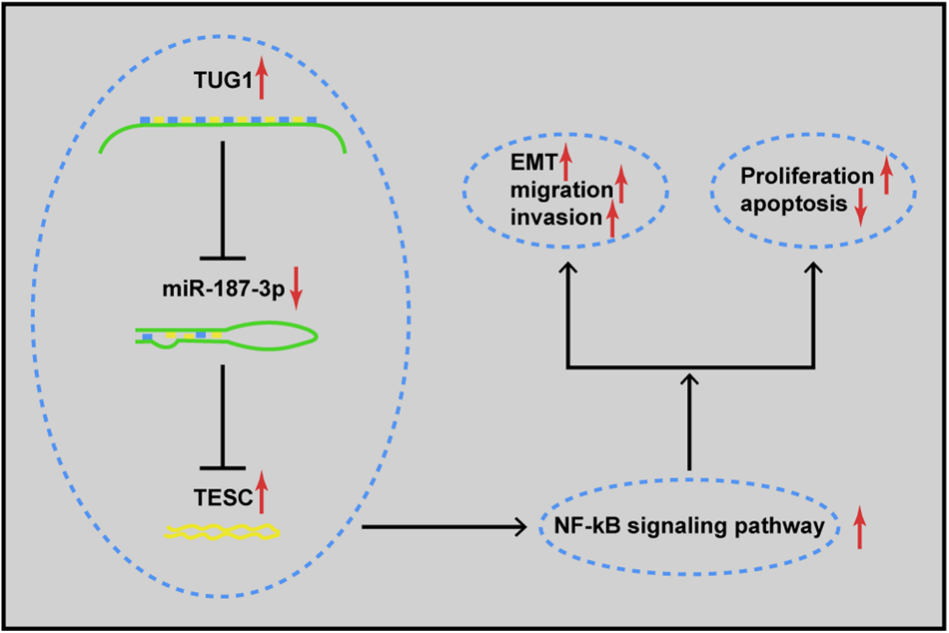
Schematic representation of a model for the major molecular mechanisms of lncRNA TUG1/miR-187-3p/ TESC axis in pituitary adenoma, which indicates that TUG1 sponges miR-187-3p to affect PA development by elevating TESC and regulating the NF-κB signaling pathway.

PA is a benign epithelial tumor that derived from the adenohypophysial cells of the pituitary gland^3^. The tumor development is regulated by gene networks, and lncRNAs may interact with miRNAs, mRNAs, or other molecules. The ceRNA hypothesis indicates that lncRNAs can act as ceRNAs to interact with miRNAs by miRNA response elements, thereby regulating the mRNA expression^34^. The present study aims to investigate the impact of the lncRNA TUG1/miR-187-3p/TESC axis on the progression of PA, and we have found that lncRNA TUG1 was able to sponge miR-187-3p to modulate the development of PA by targeting TESC and regulating the NF-κB signaling pathway (Fig. 8).

We firstly measured the expression levels of TUG1, miR-187-3p, TESC, and NF-κB pathway-related proteins, and the results suggested that TUG1 and TESC were highly expressed and the NF-κB signaling pathway was activated, while miR-187-3p was downregulated in PA tissues and cell lines. Similarly, Long et al.^12^ have revealed that TUG1 is overexpressed in melanoma specimens and cell lines, and it has been demonstrated that TUG1 is upregulated in glioma tissues^13,14^. Moreover, Liu et al. have discovered that miR-187-3p expression is inhibited in infantile hemangioma tissues^18^, and a recent document has pointed out that miR-187-3p is downregulated in HCC tissues and cell lines^19^. As for the abnormal expression of TESC, Luo et al.^23^ have found that contrasted to the normal tissues, TESC expression is elevated in RCC tissues, and it has been unraveled that the level of TESC is heightened in the tissues and serum from CRC patients^24^. Furthermore, Lu et al.^26^ have figured out that the expression of NF-κB is amplified in HP75 cells. These data indicated the abnormal expression of miR-187-3p, TUG1, and TESC, as well as the activated NF-κB signaling pathway in various tumors, providing a theoretical basis for our study. We have also clarified the regulatory relation between TUG1 and miR-187-3p, and the target relation between miR-187-3p and TESC in PA, which have not been clearly identified before.

Another vital finding in our study reflected that the expression of TUG1 was negatively related to the prognosis of PA patients. Consistently, Wei et al.^35^ have proposed that higher expression of TUG1 indicates a worse prognosis of patients with cervical cancer, and it has also been testified that TUG1 predicts a poor prognosis of prostate cancer patients^36^. These documents suit well with our finding that TUG1 acted as a predictive role in tumor diagnosis and could be used as a diagnostic biomarker. In addition, evidence in our research revealed that the inhibited TUG1 and elevated miR-187-3p were able to restrain the growth of PA cells. In line with this result, Hui et al.^37^ have verified that TUG1 inhibition could block the cell cycle and suppress the proliferation of pancreatic cancer cells, and it has been demonstrated that TUG1 facilitates the proliferation and stemness of ovarian cancer cells^38^. As for the role of miR-187-3p, Chen et al.^39^ have figured out that the overexpressed miR-187 has the ability to repress proliferation of gastric cancer cells and arrest the gastric cancer cells at the G0/G1 phase. Another publication has indicated that miR-187-3p reduces the cell viability in infantile hemangioma^18^. Additionally, we have found that the downregulation of TUG1 and upregulation of miR-187-3p could restrict the migration and invasion of PA cells. Consistently, Guo et al. have unraveled that the inhibition of TUG1 could repress the migration and invasion of bladder cancer cells^40^, and TUG1 has been revealed to promote the proliferation, migration, and invasion of HCC cells^41^. A former literature has unearthed that miR-187-3p mimic could inhibit the migration and invasion of non-small-cell lung cancer (NSCLC) cells^42^. Moreover, the promotive effects of reduced TUG1 and elevated miR-187-3p on apoptosis of PA cells have been illuminated in our research as well. A similar outcome has also been drawn out that the repression of TUG1 has the capacity to accelerate apoptosis of RCC cells^43^ and esophageal cancer cells^44^, and Sun et al.^42^ have clarified that miR-187-3p elevation promotes the apoptosis of NSCLC cells. Except for that, we have also illustrated that the knockdown of TUG1 and elevation of miR-187-3p were able to suppress tumor growth of PA in vivo. Consistent with this result, it has been recently identified that the inhibited TUG1 could restrain the tumor growth of melanoma in vivo^12^, and Cui et al.^45^ have demonstrated that miR-187 could inhibit tumor growth in osteosarcoma. The above data implied the oncogenic role of TUG1 as well as the anti-tumor role of miR-187-3p during the progression of tumors, helping us to identifying our findings.

Altogether, we have testified that the inhibited TUG1 and elevated miR-187-3p were able to decelerate the growth of both PA cells and tumors by reducing TESC, thereby repressing the progression of PA, which may be helpful for investigation on novel treatment for PA. Nevertheless, the role of TESC as well as its relation to the NF-κB signaling pathway in PA was not fully explored here. We would explain the detailed molecular mechanisms in our future works.
